# College and Hospital Notes

**Published:** 1910-03

**Authors:** 


### COLLEGE AND HOSPITAL NOTES.

---

The Buffalo General Hospital will soon establish a free maternity ward, sufficient funds having been raised by the women of the hospital board to justify this step. Announcement to this effect was made at the monthly meeting of the board of trustees, held February 24, 1910. The need for such a ward free in every sense, has long been felt and the Buffalo General Hospital trustees took steps some time ago to establish such a charity, equipped with every convenience and all the special appliances so necessary in this branch of work. To meet the requirements a considerable sum of money was needed and the women of the board undertook to finance the new department. That they have succeeded is indicated by the announcement referred to, that the ward will be immediately installed and will be in charge of Dr. P. W. Van Peyma, who will have the aid of special assistants and a corps of nurses, who have received thorough training in maternity cases at the Sloan Maternity, New York.

---

The trustees of the Buffalo General Hospital at a meeting held January 26, 1910, appointed Dr. George A. Himmelsbach and Dr. N. G. Russell assistant attending physicians, and Dr. Thew Wright, assistant attending surgeon.

---

The University of Buffalo, as has been its custom for many years, held public exercises University Day, February 22, 1910, at the Teck Theater. Addresses were made by Mayor L. P. Fuhrmann and by President John H. Finley, of the University of the city of New York. Honorable Charles P. Norton, Chancellor of the University, presided and around him on the stage sat members of the faculty, the board of trustees and city and county officials. The program opened with the singing of Alma Mater which was given with vigor.

The Mayor, in a brief speech announced his belief in a greater University of Buffalo, which he thought would play a large part in making this a greater and better city.

Dr. Finley, in an admirable address, reviewed the University situation, expressing the hope that Buffalo might have a college where the sons of the rich and poor alike should have the opportunity to obtain a college education. Such meetings serve to advance the cause of a greater University for Buffalo.

